# Dr. M. A. Veeder

**Published:** 1915-12

**Authors:** 


**Dr. M. A. Veeder,** Buffalo 1883, died at his home in Lyons November 16, aged 67. He was born at Ashtabula November 2, 1848, graduated at Union College 1879, and studied in Leipzic before entering the University of Buffalo. After graduating in medicine, he began practice in Lyons, with Dr. E. W. Bottume. Beside his affiliation with the regular medical organizations, he was a member of the American Society of Microscopists of which he had been Vice-President, and of the American Association for the Advancement of Science. As a young man, he was Principal of Ives Seminary of Antwerp, N. Y. While essentially a practitioner, he was greatly interested in the scientific aspects of medicine. He first definitely established the conveyance of typhoid by ice and by flies and was largely instrumental in the adoption of the open air treatment of tuberculosis.

